# Predictors of screening for cervical and colorectal cancer in women 50–65 years old in a multi-ethnic population

**DOI:** 10.1016/j.pmedr.2021.101375

**Published:** 2021-04-19

**Authors:** Diane M. Harper, Melissa Plegue, Ananda Sen, Sherri Sheinfeld Gorin, Mas Jimbo, Minal R. Patel, Ken Resnicow

**Affiliations:** a Department of Family Medicine, University of Michigan, Ann Arbor, MI, USA; b Department of Obstetrics and Gynecology, University of Michigan, Ann Arbor, MI, USA; c Department of Women’s and Gender Studies, University of Michigan, Ann Arbor, MI, USA; d Department of Biostatistics, University of Michigan, Ann Arbor, MI, USA; e Department of Health Behavior and Health Education, University of Michigan, Ann Arbor, MI, USA

**Keywords:** aOR, adjusted odds ratio, BMI, body mass index, COVID-19, coronavirus -19 or SARS-CoV-2 – severe acute respiratory syndrome coronavirus 2, CRC, colorectal cancer, FDA, Food and Drug Administration of the United States, FIT, fecal immunochemical test, FIT DNA, multitarget stool DNA test, FOBT, fecal occult blood test, MENA, Middle Eastern/North African, MT, sDNA test – multitarget stool DNA test - Cologuard®, OR, odds ratio, SE, standard error, USPSTF, United States Preventive Services Task Force, Cervical cancer screening, Colorectal cancer screening, Females, 50–65 years old, Middle Eastern-North American (MENA) ethnicity

## Abstract

Middle Eastern/North Africa (MENA) women are often not identified in cancer screening studies. The aim of this study was to determine the rates and predictors of cervical and colorectal cancer (CRC) screening for women 50–65 years of three race/ethnicities.

White, black and MENA women of Southeast Michigan were surveyed once in 2019 for demographics, health care barriers, chronic diseases, and cancer screening updates using in-person, telephone, and online methods. Descriptive statistics and multivariate multinomial logistic regression were used to predict up-to-date colorectal cancer and cervical cancer screening. All analyses were adjusted by local population weights for comparability and generalizability.

394 women participated with 54% up-to-date on both screenings, 21% for cervical cancer screening alone, and 12% for CRC alone. Women more likely to be up-to-date for only cervical cancer screening compared to both cancer screens are younger (aOR 0.83 (95% CI 0.76, 0.92), are of MENA descent (7.97 (2.46, 25.76) and have no insurance (9.41 (1.07, 82.92). There are no predictors for women being up-to-date for CRC screening alone compared to both screens.

Among women 50–65 years old, being up-to-date in cervical cancer screening is unrelated to being up-to-date for CRC screening. Compared to Healthy People 2020, there are significant gaps in cervical and CRC screening among women 50–65 years old of all races, but particularly among women of MENA descent who are even less likely to have CRC screening than cervical cancer screening.

## Highlights

• Middle Eastern/North African (MENA) women in the US have the lowest up-to-date screening rate of colorectal and cervical cancer of all races/ethnicities at 18%.
• Any chronic health condition doubles the likelihood of having both below the belt screens compared to neither screen.
• Women without insurance are more likely to only have cervical cancer compared to both colorectal and cervical cancer up-to-date screens.
• 54% of women 50–65 years old are up-to-date with both colorectal and cervical cancer screening.

## 1 Introduction

Colorectal and cervical cancers are the ‘below the belt’ cancers for which screening rates among US women 50–65 years old are lower than the Healthy People 2030 targets of 74.4% and 84.3%, respectively (Office of Disease Prevention and Health Promotion. (n.d.), 2030). To date, screening rates in this age group are 65.4% (95%CI: 64.5, 66.2) for colorectal cancer (CRC) and 74.1% (95% CI: 71.7, 76.5) for cervical cancer (Joseph et al., 2020). Much of the screening gap occurs in the 50–65-year-old age group (American Cancer Society, 2019) and is related to not having health insurance, limited education, and living in a rural area (Joseph et al., 2020, Harper et al., 2020, Shi et al., 2011). Menopause, chronic diseases, partner, and employment changes may also impact the lower rates for many racial groups (Munnell et al., 2019, Aftab et al., 2019, Rix, 2012, Kreider and Ellis, 2009). There are no studies, though, reporting the prevalence of MENA cervical or colorectal cancer screening in the US.

Both colorectal and cervical cancer are amenable to home self-screening. Home testing provides privacy and a way to overcome the embarrassment often associated with the tests. (Ritvo et al., 2013). Home testing provides convenience and access for those living in rural areas. Currently, 72% of initial CRC screens are done by stool-based testing (Wernli et al., 2014) with one of the following methods: the fecal occult blood test (FOBT), fecal immunochemical test (FIT), and multitarget stool DNA test (MT-sDNA test, or FIT-DNA) all of which are FDA approved, standard of care, and recommended specifically as a home test by the United States Preventive Services Task Force (USPSTF) (Bibbins-Domingo et al., 2016, Gupta et al., 2020). Cervical cancer screening through a primary HPV test is being fielded around the world as a means of reaching women who otherwise would not be screened (Caleia et al., 2020, Sahasrabuddhe et al., 2011), and home self -sampling kits are under evaluation for Food and Drug Administration (FDA) approval. In addition, we have seen the disruption in both cancer screening preventive services with an 86% decline in colorectal and 94% decline in cervical cancer screening nationally due to the coronavirus-19 (COVID-19) pandemic (Health, 2020). In a study of the impact of COVID-19-related clinic closures on cancer screening in a large midwestern academic medical center, screening colonoscopies declined 99.9%, but MT-sDNA testing (via Cologuard®) only declined 65% between January 19, 2020 and April 19, 2020 (SheinfeldGorin et al., 2020). This finding suggests that adults were willing and able to complete cancer screenings at home when offered the option.

Women have different predictors of CRC screening than men (Brittain et al., 2012), as well as lower overall screening rates (Wernli et al., 2014). Women who are up-to-date for CRC are also more likely to be up-to-date with cervical cancer screening (Wirth et al., 2014), to have a higher educational attainment and be employed (McGregor and Bryant, 2005). However, we do not know the predictors for completing both CRC and cervical cancer screening in women aged 50–65 years. It is uncertain how the predictors of screening change when the screening is both CRC and cervical cancer compared to a single cancer or neither cancer.

These two cancers are uniquely combined together for this study because past work has shown that general internists are significantly less likely to offer cervical cancer screening to women older than 30 years compared to family physicians or gynecologists (Corbelli et al., 2014), whereas general internists are more likely to recommend CRC screening than family physicians (Higgins et al., 2012). Depending on her primary care physician, the woman may or may not be invited to screen equally for both cancers.

The primary aim of this work is to determine the predictors of both up-to-date cervical and CRC screening in three races/ethnicities of women in Southeast Michigan who are 50–65 years old. The study is unique in that it includes MENA women, a group rarely included in population studies. The secondary aim of this work is to evaluate screening prevalences for cervical and/or colorectal cancer among 50–65 years old women.

## 2 Methods

### 2.1 Survey

A community-based cross-sectional health survey was developed and piloted to query the southeast Michigan populations of white, black, and of MENA descent (Resnicow et al., 2020). The catchment area is white majority (80–97%), Black (5–22%) and is home to the highest MENA population east of the Mississippi. The broader study included men and women ages 18–80 (supplement). For this study we included only women 50–65 years of age. Demographic descriptors included age within study range, race/ethnicity, birthplace, education, income, marital status, occupational status, and body mass index (BMI). Barriers assessed were transportation and lack of insurance. Co-morbid chronic conditions assessed were diabetes, hypertension, cardiac diseases, lung diseases, arthritis, depression, and a past cancer diagnosis. Those who ever had cervical or colon cancer were removed from the database as exclusion criteria. The survey did not assess whether women had a prior hysterectomy.

The outcome variables of interest were up-to-date cancer screenings for cervical cancer and colorectal cancer. We defined up-to-date as having had a cervical cancer screen (Pap test) within the past 3 years and did not include HPV testing status. For colorectal cancer, we defined up-to-date to be having an FOBT within the past year or a colonoscopy within the past 10 years in accordance with the USPSTF guidelines (Bibbins-Domingo et al., 2016). No other methods of CRC screening were queried on the survey.

### 2.2 Survey administration

Between May 1 through October 28, 2019, three approaches were used to recruit and administer the survey: (1) a landline or cell phone random dial phone interview resulting in 496 participants, (2) online questionnaire to a panel of respondents resulting in 1122 participants and (3) an in-person/phone/mail/online survey of MENA communities in southeast Michigan resulting in 214 participants (Resnicow et al., 2020). A landline or cellphone random dial phone interview conducted by Harris Interactive Inc. sampled White/Caucasian and Black/African American adults the online sample was recruited through Dynata (www.dynata.com) which oversampled African Americans in the catchment area and had a 9% response rate. Whereas the community sample was a convenience sample, the telephone and online sample was built to match the demographic representation of the University of Michigan Rogel Cancer Center catchment area.

Finally, a survey designed for the MENA population was distributed at 12 diverse settings of MENA interest across three southeast Michigan counties. The MENA women could complete the survey using paper and pen or electronic on-line forms (with a provided tablet device), with or without assistance, in English or Arabic, at their home (pre-stamped return envelope or web address), or by phone. Data collectors were fluent in English and Arabic. All participants from all three approaches received an incentive for survey completion.

## 3 Statistical analyses

There were 394 participants in our study. Participants were categorized into one of four outcome groups based on screening behavior: up-to-date for both cervical and colorectal cancer screens, up-to-date on cervical cancer screening only, up-to-date on colorectal cancer screening only or not up-to-date on either. Descriptive statistics including means and frequencies were calculated for all variables across the four groups. One-way analysis of variance assessed differences in age across groups with post-hoc pairwise comparisons. Chi-square tests were used for comparing distributions of categorical variables across groups. Multivariable multinomial logistic regression modeling was used for the complete case analysis to determine predictors of up-to-date cervical and/or colorectal cancer screening using variables determined to be important based on prior research and bivariate analysis.

Sampling weights were created for each observation across the three surveys. Post-stratification weight adjustment for the Harris and Dynata survey samples were done by calculating population totals in the geographic region of interest using the 2017 American Community Survey. A 3-dimensional raking approach was implemented using demographic variables of age, gender, and race/ethnicity. The MENA sample was weighted using one-dimensional post-stratification on mother and father’s country of origin; population totals used came from estimates created by the Arab-American Institute for the geographic area (Institute et al., 2018). In the presence of missing data on variables used for post-stratification, imputation using the probability distributions of non-missing values was used. Final weights were assessed by comparing weighted sums of demographic variables to original population totals in each post-stratification cell. All analyses were adjusted for survey weights to allow interpretations of results at the Southeast Michigan population level. Statistical analysis was done using Stata version 15.1 (StataCorp., 2017). This study was approved by Protocol #: HUM00159558 approved March 10, 2019.

## 4 Results

There were 394 women who met the study inclusion criteria; 54% were up-to-date for both a cervical cancer and a CRC screen, 13% had neither; 21% were up-to-date on their cervical cancer screen alone, and 12% were up-to-date with a CRC screen alone. Among those with an up-to-date CRC screen, 93% had a colonoscopy; and 7% had FOBT.

The demographic descriptors, barriers and comorbid chronic diseases of the study population are shown as weighted results in Table 1. Age is significantly different among the screening groups (F (3,298) = 11.9, p < 0.001) with post hoc testing showing women with both screens up-to-date being older, on average, than those with only cervical cancer screening (mean(SE) = 58.8(0.3) vs 55.5(0.6), p-value < 0.001) and those with neither screen (mean(SE) = 58.8(0.3) vs 56.0(0.8), p-value = 0.013). Women with only CRC screening were significantly older, on average, than women with only cervical cancer screening (mean(SE) = 59.8(0.7) vs 55.5(0.6), p < 0.001) and women with neither screen (mean(SE) = 59.8(0.7) vs 56.0 (0.8), p = 0.007).

**Table 1:** Demographic descriptors of women 50–65 years old **by currency** with cervical and colorectal cancer screenings.

	Both cervical cancer and CRC screening		Cervical cancer screening alone		CRC screening alone		Neither screening		
	n=213 (54%)		n=82 (21%)		n=47 (12%)		n=52 (13%)		p-value
Age in years Unweighted, mean(SD) Weighted, mean(SE)	58.4 (4.3) 58.8 (0.31)		55.7 (4.6) 55.5 (0.61)		59.1 (4.4) 59.8 (0.71)		56.5 (4.1) 56.0 (0.79)		<0.001
	N (%)	Weighted % (95% CI)	N (%)	Weighted % (95% CI)	N (%)	Weighted % (95% CI)	N (%)	Weighted % (95% CI)	p-value
Race (n, row %)									0.007
White	157 (58%)	63% (55.5, 69.0)	46 (17%)	15% (10.5, 20.3)	36 (13%)	14% (9.8, 19.7)	32 (12%)	9% (5.5, 13.4)	
Black	42 (56%)	54% (40.5, 67.2)	15 (20%)	17% (9.8, 28.6)	8 (11%)	12% (5.1, 25.3)	10 (13%)	17% (9.1, 28.8)	
Middle Eastern North African (MENA)	8 (22%)	18% (8.7, 32.5)	19 (51%)	55% (37.9, 71.8)	2 (5%)	7% (1.5, 27.7)	8 (22%)	20% (9.4, 37.0)	
Other ^a^	6 (55%)	64% (22.6, 91.5)	2 (18%)	18% (2.4, 66.7)	1 (9%)	0%	2 (18%)	18% (2.4, 66.7)	
Born in United States (n, row %)									0.42
Yes	199 (57%)	61% (54.6, 67.2)	62 (18%)	16% (11.4, 20.7)	43 (12%)	14% (9.6, 18.8)	44 (13%)	10% (6.7, 14.3)	
No	14 (30%)	52% (32.3, 71.2)	20 (43%)	28% (15.5, 44.4)	4 (9%)	11% (2.6, 35.3)	8 (17%)	9% (4.1, 20.3)	
Education (n, row %)									0.05
High School or less	39 (46%)	56% (42.5, 69.2)	20 (24%)	16% (8.6, 25.8)	7 (8%)	10% (4.0, 23.6)	19 (22%)	18% (10.2, 30.6)	
Some College	63 (51%)	58% (46.5, 68.6)	30 (24%)	19% (12.3, 29.9)	14 (11%)	11% (5.6, 20.8)	16 (13%)	11% (5.9, 20.7)	
College graduate	85 (62%)	66% (56.3, 75.3)	27 (20%)	17% (10.5, 25.8)	14 (10%)	11% (6.2, 19.4)	12 (9%)	5% (2.4, 12.0)	
Post college education	26 (54%)	54% (36.9, 70.8)	5 (10%)	8% (2.9, 21.1)	12 (25%)	30% (16.1, 48.5)	5 (10%)	8% (2.2, 22.8)	
Income (n, row %)									0.27
<$10*K*	11 (39%)	37% (18.2, 60.5)	9 (32%)	21% (9.1, 41.9)	2 (7%)	17% (4.6, 48.1)	6 (21%)	25% (9.6, 50.0)	
$10-$49999	68 (46%)	53% (42.9, 63.6)	37 (25%)	21% (14.2, 31.1)	19 (13%)	14% (7.7, 22.6)	24 (16%)	12% (6.6, 19.7)	
$50-$99999	86 (61%)	67% (56.6, 75.7)	24 (17%)	12% (6.9, 19.6)	16 (11%)	13% (7.1, 21.4)	14 (10%)	9% (4.5, 16.3)	
≥$100,000	44 (63%)	64% (49.9, 76.4)	12 (17%)	16% (8.4, 28.9)	10 (14%)	15% (7.3, 28.6)	4 (6%)	5% (1.1, 16.4)	
Marital Status (n, row %)									0.30
Married or partnered	132 (56%)	60% (52.0, 67.6)	43 (18%)	14% (9.7, 20.1)	33 (14%)	16% (11.1, 23.5)	28 (12%)	9% (5.7, 15.2)	
Single ^b^	81 (52%)	62% (52.1, 70.9)	38 (24%)	19% (12.2, 27.6)	14 (9%)	9% (4.5, 16.2)	23 (15%)	11% (6.3, 17.6)	
Occupational Status (n, row %)									<0.001
Employed	109 (56%)	61% (51.9, 68.7)	46 (23%)	22% (15.5, 29.6)	15 (8%)	7% (4.0, 13.8)	26 (13%)	10% (6.1, 16.3)	
Unemployed	10 (38%)	39% (18.6, 64.1)	10 (38%)	40% (19.7, 64.0)	1 (4%)	9% (1.3, 42.8)	5 (19%)	12% (3.5, 35.2)	
Homemaker	16 (46%)	51% (28.9, 72.7)	12 (34%)	18% (6.9, 39.5)	3 (9%)	20% (6.6, 46.1)	4 (11%)	11% (3.1, 33.7)	
Retired	53 (58%)	64% (51.2, 75.3)	9 (10%)	6% (2.1, 14.0)	21 (23%)	26% (16.3, 38.7)	8 (9%)	4% (1.4, 13.4)	
Disabled	21 (55%)	64% (45.2, 79.9)	5 (13%)	5% (0.9, 19.2)	5 (13%)	11% (3.5, 29.1)	7 (18%)	20% (9.0, 39.5)	
BMI (kg/m^2^) (n, row %)									0.59
Underweight	4 (67%)	79% (28.1, 97.4)	1 (17%)	21% (2.6, 71.9)	0	0	1 (17%)	0	
Normal	67 (59%)	68% (56.7, 78.1)	21 (19%)	12% (6.8, 22.1)	12 (11%)	11% (5.7, 21.5)	13 (12%)	8% (3.4, 16.6)	
Overweight	53 (52%)	52% (40.2, 63.8)	22 (22%)	21% (12.8, 31.4)	15 (15%)	19% (11.0, 30.5)	11 (11%)	8% (3.8, 17.8)	
Obese	87 (53%)	61% (51.2, 69.7)	32 (20%)	15% (9.4, 22.5)	20 (12%)	12% (7.0, 20.1)	24 (15%)	12% (7.4, 19.7)	
Barriers									
Transportation (n, %)									0.47
Always/Usually/Sometimes a barrier	30 (51%)	61% (45.0, 75.7)	16 (27%)	20% (9.9, 34.1)	4 (7%)	7% (1.9, 22.6)	9 (15%)	12% (5.1, 25.7)	
Rarely a barrier	25 (42%)	53% (36.0, 68.7)	17 (29%)	26% (13.8, 42.4)	9 (15%)	10% (3.6, 23.9)	8 (14%)	12% (4.8, 27.0)	
Never a barrier	157 (58%)	62% (54.5, 68.6)	47 (17%)	14% (9.8, 19.7)	34 (12%)	15% (10.5, 21.4)	34 (12%)	9% (5.7, 14.1)	
Insurance (n, row %)									0.011
Private	146 (59%)	62% (54.5, 69.4)	43 (17%)	15% (10.7, 21.6)	29 (12%)	14% (9.2, 20.3)	28 (11%)	9% (5.1, 13.8)	
Public ^c^	60 (54%)	62% (50.1, 72.0)	26 (23%)	14% (7.9, 22.4)	13 (12%)	14% (7.2, 24.1)	13 (12%)	11% (5.8, 20.5)	
None	2 (9%)	14% (2.9, 48.0)	8 (35%)	50% (23.0, 77.2)	4 (17%)	6% (1.2, 24.5)	9 (39%)	30% (11.4, 58.2)	
Chronic conditions (n, row%)									
Any	163 (56%)	62% (54.6, 68.5)	57 (20%)	16% (11.7, 22.0)	40 (14%)	15% (10.2, 20.6)	31 (11%)	7% (4.5, 11.9)	0.11
Diabetes	47 (55%)	62% (47.8, 73.7)	12 (14%)	7% (3.0, 16.3)	12 (14%)	19% (10.0, 32.0)	14 (16%)	12% (6.1, 24.1)	0.15
Hypertension	92 (58%)	64% (54.2, 72.7)	24 (15%)	13% (7.8, 20.8)	24 (15%)	15% (9.4, 23.8)	18 (11%)	8% (4.1, 14.3)	0.47
Cardiac	7 (50%)	49% (21.4, 77.8)	2 (14%)	20% (4.9, 54.3)	1 (7%)	13% (1.9, 55.3)	4 (29%)	17% (4.8, 46.9)	0.82
Lung	31 (60%)	67% (50.9, 79.6)	8 (15%)	15% (6.8, 30.1)	6 (12%)	9% (3.4, 21.3)	7 (13%)	9% (3.4, 22.8)	0.76
Arthritis	77 (54%)	61% (50.4, 70.3)	31 (22%)	17% (10.4, 25.2)	19 (13%)	13% (7.4, 22.2)	16 (11%)	10% (5.0, 17.3)	0.99
Depression	68 (55%)	57% (45.7, 66.9)	29 (23%)	20% (12.6, 29.3)	18 (15%)	17% (10.5, 27.6)	9 (7%)	6% (2.6, 14.0)	0.22
Cancer	29 (59%)	62% (45.3, 76.1)	7 (14%)	13% (5.6, 26.9)	11 (22%)	23% (11.9, 39.7)	2 (4%)	2% (0.3, 15.1)	0.11
Cancer Types, n (unweighted %)									
Bone	1	100%	0	0%	0	0%	0	0%	
Breast	10	63%	2	9%	4	28%	0	0%	
Endometrial	1	0%	0	0%	0	0%	0	0%	
Hodgkin’s	0	0%	1	100%	0	0%	0	0%	
Melanoma	3	100%	0	0%	0	0%	0	0%	
Non-Hodgkin’s	1	50%	0	0%	1	50%	0	0%	
Ovarian	3	63%	0	0%	2	21%	1	17%	
Pancreatic	0	0%	0	0%	1	0%	0	0%	
Skin	7	70%	1	15%	2	15%	0	0%	

The weighted racial distribution was 87% white, 8% black, 3% MENA and 2% other races including Asian Indian, Pacific Islander, American Indian, Alaska Native, Chinese and those of more than one race. There was an overall difference in screening patterns across the racial groups (p = 0.007). MENA women appear to screen less for both cancers and significantly more for cervical cancer screening alone than other racial groups. There did not appear to be a significant association with whether or not the woman was born in the United States (p-value = 0.42).

Screening patterns were also significantly different across occupational status (p < 0.001) with unemployed women having the highest rates of up-to-date cervical cancer screening and women with disabilities having the highest rates not being up-to-date for either cancer screening. The remaining demographic descriptors, education, income, marital status, and BMI were not differentially distributed across screening groups.

Among barriers to screening, transportation did not differ across screening groups. Insurance, on the other hand, was significantly different across the groups (p < 0.011) as women with no insurance had lower rates of both CRC and cervical cancer screening compared with those who had private and public insurance, and higher rates of neither cancer screening.

Comorbid chronic conditions and past cancers were not significantly different across screening groups.

## 5 Predictors of screening patterns

Table 2 provides a summary of the multivariable multinomial regression analyses adjusting for age, race, insurance, and presence of any chronic medical condition. While occupational status showed a significant association in the bivariate comparisons, it was removed from the final model due to a high level of correlation with age as well as a sparse distribution in some categories leading to unstable coefficient estimates. Results are provided using different reference populations for the outcome to allow for the evaluation of all possible comparisons.

**Table 2:** Multinomial Logistic Regression Model Predicting Up to Date Cancer Screening Status.

	**Referent Outcome: Neither Screen**			**Referent Outcome: Both Screens**		**Referent Outcome:Cervical Only**
	**Both Screens**	**Cervical Only**	**CRC Only**	**Cervical Only**	**CRC Only**	**CRC Only**
	**aOR (95% CI)**	**aOR (95% CI)**	**aOR (95% CI)**	**aOR (95% CI)**	**aOR (95% CI)**	**aOR (95% CI)**
**Age**	1.14 (1.03, 1.26)*	0.95 (0.84, 1.08)	1.20 (1.05, 1.38)**	0.83 (0.76, 0.92)***	1.06 (0.95, 1.17)	1.26 (1.11, 1.44)**
**Race(Ref: White)**						
Black	0.61 (0.24, 1.56)	0.50 (0.17, 1.48)	0.52 (0.13, 2.14)	0.82 (0.32, 2.11)	0.85 (0.27, 2.70)	1.04 (0.24, 4.53)
MENA	0.28 (0.07, 1.09)	2.21 (0.56, 8.68)	0.63 (0.07, 5.59)	7.97 (2.46, 25.76)**	2.26 (0.32, 15.71)	0.28 (0.03, 2.45)
**Insurance(Ref: Private)**						
Public	0.64 (0.24, 1.75)	0.49 (0.15, 1.53)	0.63 (0.18, 2.18)	0.75 (0.32, 1.77)	0.97 (0.40, 2.39)	1.29 (0.41, 4.09)
None	0.09 (0.01, 0.78)*	0.87 (0.21, 3.56)	0.18 (0.03, 1.09)	9.41 (1.07, 82.92)*	2.00 (0.23, 17.17)	0.21 (0.03, 1.50)
**Any ChronicCondition**	2.64 (1.04, 6.66)*	2.98 (1.03, 8.58)*	3.51 (1.01, 12.19)*	1.13 (0.46, 2.76)	1.33 (0.47, 3.74)	1.18 (0.35, 3.99)

*p-value < 0.05

**p-value < 0.01

***p-value < 0.001

aOR means odds ratio adjusted for age, race, insurance, and any chronic condition (at least one of diabetes, hypertension, heart disease, lung disease, arthritis, depression or prior non-colon, non-cervix cancer)

Using the comparator of ***neither colorectal nor cervical cancer screening***, it was found that having at least one chronic comorbid condition resulted in higher odds of being up-to-date with one (Cervical aOR(95% CI) = 2.98 (1.03, 8.6) and CRC 3.51 (1.01, 12.2)) or both (aOR(95% CI) = 2.64 (1.04, 6.7)) cancer screens. Increased age also led to significantly higher odds of being up-to-date for CRC alone (aOR(95% CI) = 1.2 (1.05, 1.4)) and both screens (aOR(95% CI) = 1.14 (1.03, 1.3)) but did not have an effect on cervical screen only by comparison to neither screen up-to-date. Uninsured individuals had 91% lower odds (aOR (95% CI) = 0.09 (0.01, 0.78)) than those who had private insurance of being up-to-date on both screens compared with neither.

When compared to being up-to-date for ***both*** cervical and colorectal cancer screening, age, race and being uninsured were predictive of being up-to-date on cervical cancer screening only. Older individuals were less likely to be screened for cervical cancer only (aOR (95% CI) = 0.83 (0.8, 0.9)). Women of MENA ethnicity had odds 8 times higher than white women of being screened for only cervical cancer (aOR (95% CI) = 7.97 (2.5, 25.8)). Uninsured women also had much higher odds than the privately insured women of having only cervical cancer screening (aOR(95% CI) = 9.4 (1.1, 82.9)).

The only significant predictor when comparing between single screens was age, where older individuals had higher odds of being screened for CRC compared to cervical cancer alone (aOR(95% CI) = 1.26 (1.1, 1.4)).

## 6 Discussion

We are the first to publish data on the uptake of CRC and cervical cancer screening among women 50–65 years old in southeast Michigan. We also are the first to show that MENA women are unscreened for CRC cancer even more than for cervical cancer. We show for women 50–65 years old that there are different predictors for completing both colorectal and cervical cancer screening compared to either of the two single screens or no screen at all. Our data align with other studies (Wirth et al., 2014) which show that a portion of women who are up-to-date with colorectal cancer screening are also up-to-date with cervical cancer screening. Over half (54%) of the women in our study were current in both colorectal and cervical cancer screening, with 75% current for cervical and 66% for colorectal cancer screenings, still below the HP2020 (Office of Disease Prevention and Health Promotion, 2030) goal for either cancer alone.

Race/ethnicity is usually a predictor for up-to-date cancer screenings. Only the MENA race/ethnicity was a significant predictor when we considered being up-to-date for two screens. This has large public health implications for future community outreach to MENA women who have not yet benefited from combined colorectal and cervical cancer screening. While studies have shown a lack of cervical cancer screening due to cultural barriers (Padela and Rodriguez del Pozo, 2011) in women of MENA descent, no work has addressed the same cultural barriers to colorectal cancer screening or in the combination of screens.

Traditional positive binary predictors of cervical cancer screening (yes/no) in this age group have included younger ages, higher educational attainment, higher income, and Medicaid insurance in addition to urban residence, and Black and Hispanic races (Harper et al., 2020, Shi et al., 2011). Barriers to screening have included Asian race, being uninsured, low education, low income, and not being married (Harper et al., 2020, Shi et al., 2011).

Traditional positive binary predictors for colorectal cancer screening (yes/no) include increasing age, higher educational attainment, higher income, private insurance (Zhang et al., 2020, Shi et al., 2011, Ioannou et al., 2003 Sep). Barriers to screening have included Hispanic race, public insurance, no insurance, the 50–64 year old age group itself, less education, and low income (Zhang et al., 2020, Shi et al., 2011, Ioannou et al., 2003 Sep).

In our study, we showed that the predictors of up-to-date screening for two cancers are different than the predictors of screening for a single cancer. This is important because as middle aged women age into menopause through 65 year of age, her primary care physician has the opportunity for person-centered health care that involves both ‘below the belt’ cancer screens. Compared to a single cancer screen, age, race, insurance, and at least one chronic health condition are predictors of combined colorectal and cervical cancer screening, but in different ways.

Having at least one chronic disease such as hypertension, diabetes, heart disease, lung disease, arthritis, or depression differentiated women in our study up-to-date with one or both screens from those who were not up-to-date with either screen. Other work shows that having depression was associated with higher up-to-date CRC screening rates (Petrik et al., 2018); but having at least one of eleven chronic conditions led to lower up-to-date cervical cancer screening rates (Crawford et al., 2016). Our results suggest that a woman with a chronic disease would have increased opportunity to participate in both ‘below the belt’ cancer screens.

Age is usually a predictor for up-to-date cancer screenings. And ages 50–65 are of particular importance for women. Colorectal cancers have continued to increase in women 50–65 years old and currently make up a third of all the colorectal cancer diagnoses, with recommendations to start screening earlier at 45 years (Siegel et al., 2020); while at the same time**;** one third of all cervical cancers are detected in women 50–65 years old (Quinn et al., 2019), and at more advanced stages. A more person-centered approach to ‘below the belt’ cancer screenings could address both screens, especially if both screens could be performed at home (SheinfeldGorin et al., 2020).

Insurance status remains a strong predictor for both up-to-date CRC and cervical cancer screening as well as cervical cancer screening alone. While the Accountable Care Act mandated zero out of pocket costs for these preventive services, women would have had to sign up for a public insurance program to benefit. The lack of insurance coverage reflects the economic status of women 50–65 years in non-traditional workplaces where private insurance is not available (Munnell et al., 2019) and public insurance still has a cost. The patient’s in-office cost of colonoscopy screening, that is frequent in this sample as well as nationally, is significantly higher than cervical cancer screening both in terms of bowel prep time and procedural costs. In addition, should a cancer be detected, the cost of treatment could be unaffordable (WHO, 2017). Insurance has been removed as a barrier from cervical cancer screening, in part, because of the Breast and Cervical Cancer Early Detection and Prevention screening program (NBCCEDP., 2020) for those without insurance. The similar CRC CDC program, Colorectal Cancer Screening Program, (CRCCP., 2020)on the other hand, while available in Michigan, may not have had widespread implementation of its program, especially to the MENA population, hence leaving unbalanced public options for CRC vs cervical cancer screening.

Physicians have not packaged ‘below the belt’ cancer screening for women in a comprehensive manner. Reasons to initiate home based dual screening include convenience and privacy. We know that women with past sexual abuse may reject speculum based pelvic exams and colonoscopy, as they may stir unwanted memories (Wolf, 2006, Güneş and Karaçam, 2017). Near future changes in community and physician education must include addressing both below the belt cancers rather than each singly. Current work is ongoing for FDA approval of HPV testing by self-sampling methods, so that home-based sampling will be available for cervical cancer screening. COVID showed us that CRC home based screenings were actively occurring when all face to face care stopped (SheinfeldGorin et al., 2020), leading us to conclude that patient-centered, less invasive screening can be an option for both cancers for women 50–65 years old.

## 7 Limitations

The limitations of this study include a cross-sectional survey that does not allow causation to be evaluated. Only one set of survey findings was collected using a probability sample; the survey response rates were relatively low. With three survey approaches, however, we were able to capture a diverse sample of MENA, who have been little studied for cancer control. We can calculate predictors but not reasons why the predictors are associated with the outcome screenings. Likewise, all outcomes were self-reported with the opportunity to over-estimate actual screening frequencies (Ferrante et al., 2008). An overestimation of up-to-date screenings creates a greater need to develop ‘below the belt’ strategies for both cervical and colorectal cancer screenings.

## 8 Conclusions

We have identified the gap in below the belt dual cancer screenings for CRC and cervical cancers among women 50–65 years old. In addition, we have identified the dual cancer screening gap for MENA women. Population weighted prevalences of up-to-date dual screening for CRC and cervical cancer show that women of MENA descent have significantly less dual screening than any other race. Future research will develop interventions to improve both screening rates among underserved women.

## Funding

This work was supported by the National Cancer Institute Grant P30CA046592-29-S4 and the National Center for Advancing Translational Science Grant UL1TR001070.

## CRediT authorship contribution statement

**Diane M. Harper:** Conceptualization, Methodology, Resources, Writing - original draft. **Melissa Plegue:** Software, Formal analysis, Writing - review & editing. **Ananda Sen:** Conceptualization, Writing - review & editing. **Sherri Sheinfeld Gorin:** Conceptualization, Writing - review & editing. **Mas Jimbo:** Conceptualization, Writing - review & editing. **Minal R. Patel:** Conceptualization, Investigation, Writing - review & editing. **Ken Resnicow:** Conceptualization, Methodology, Investigation, Resources, Writing - review & editing.

## Declaration of Competing Interest

The authors declare that they have no known competing financial interests or personal relationships that could have appeared to influence the work reported in this paper.
